# Relationship between stress and coronary artery disease: A comprehensive review

**DOI:** 10.1097/MD.0000000000037066

**Published:** 2023-02-02

**Authors:** Chukwuka Elendu, Dependable C. Amaechi, Tochi C. Elendu, Klein A. Jingwa, Osinachi K. Okoye, Border-ere Fiemotonghan, Grecia A. Chirinos, Deborah Agada, Minichimso John Okah, Opeyemi D. Adebayo, Kanishk Dang, Emmanuel Egbunu, Omotayo S. Alabi, Vaibhav S. Nasre, Cyrus P. Yadav, Muhydeen D. Badru

**Affiliations:** a University of California, Santa Cruz, CA; b Igbinedion University, Okada, Nigeria; c Imo State University, Owerri, Nigeria; d Kazan State Medical University, Kazan, Russia; e Chukwuemeka Odumegwu Ojukwu University Teaching Hospital, Awka, Nigeria; f Jackson State University, Jackson, MS; g Lisandro Alvarado Centroccidental University, Barquisimeto, Venezuela; h Babcock University, Ilishan-Remo, Nigeria; i Nicolae Testemițanu State University of Medicine and Pharmacy, Chişinău, Republic of Moldova; j Federal Medical Centre, Bida, Nigeria; k Osun State University, Osogbo, Nigeria; l Obafemi Awolowo University, Ile-Ife, Nigeria.

**Keywords:** comparative analysis, coronary artery disease, environmental factors, genetic loci, literature review, pathophysiological mechanisms, stress

## Abstract

Coronary artery disease (CAD) poses a substantial public health challenge. This review examines the intricate relationship between psychological stress and CAD, drawing from recent research spanning the last 5 to 10 years. The literature review is organized around critical themes. It includes an analysis of genetic loci in CAD susceptibility and underscores the role of green environments in reducing cardiovascular risk. A quantitative analysis presents numerical findings for clarity, while pathophysiological mechanisms are elucidated through informative figures and diagrams. The review engages with controversies and disparities in the literature, offering a balanced perspective. A tabular comparative analysis outlines the strengths and limitations of existing approaches, emphasizing conflicting findings, and environmental factors. The review concludes by distilling key takeaways for healthcare professionals and researchers. Practical implications are explored, and lessons learned from the research process are reflected upon. The conclusion also suggests avenues for further study in understanding stress’s impact on CAD.

## 1. Introduction

Coronary artery disease (CAD) remains a pervasive and life-altering cardiovascular condition, posing a considerable global health burden. The intricate relationship between CAD and psychological stress has garnered substantial attention in recent years. Stress, stemming from various sources, such as work-related pressures, financial concerns, and personal life events, can manifest as a pivotal factor in the development and progression of CAD. Understanding this association is of paramount importance in modern healthcare.

Over the past decade, research in the field of CAD and stress has significantly expanded, revealing a multifaceted connection. The pathophysiological mechanisms through which stress impacts CAD risk are increasingly elucidated, encompassing inflammatory responses, autonomic dysregulation, and endothelial dysfunction.^[1]^ Genetic factors also play a role, with certain loci conferring susceptibility to CAD under stress conditions.^[2]^ Additionally, environmental factors have gained recognition for their protective effects, with green environments associated with a lower risk of cardiovascular diseases.^[3]^

However, as the body of knowledge continues to grow, so too does the complexity of this relationship. Controversies and conflicting findings have emerged, necessitating a careful examination of the literature. This review aims to address these challenges and provide a comprehensive overview of the current state of knowledge. By delving into recent research and offering a comparative analysis of different approaches,^[4]^ this review aims to provide a nuanced perspective on the connection between stress and CAD.

In a time when the impacts of stress on cardiovascular health are more significant than ever, it is vital to explore these complexities. This review aspires to shed light on the topic, ultimately contributing to a deeper understanding of the interplay between stress and CAD.

## 2. Literature review

The literature review is structured into distinct categories, each exploring various facets of the intricate relationship between stress and CAD. Within the last 5 to 10 years, a plethora of research has contributed to our understanding of this critical topic.

### 2.1. Pathophysiological mechanisms

The pathophysiological mechanisms connecting stress and CAD have been an active research area. Recent studies have highlighted the role of inflammation in this association.^[1]^ Chronic psychological stress can lead to elevated levels of pro-inflammatory cytokines, which, in turn, promote atherosclerosis and CAD.^[2]^ Autonomic dysregulation, as seen in chronic stress, contributes to increased sympathetic activity and reduced parasympathetic activity, resulting in unfavorable changes in heart rate variability and blood pressure.^[3]^ Moreover, endothelial dysfunction, linked to oxidative stress, has been recognized as a key player in the pathogenesis of CAD under stress conditions.^[4]^

### 2.2. Genetic susceptibility

Understanding genetic factors predisposing individuals to CAD in stress is a growing area of research. Recent genetic studies have identified specific loci associated with CAD susceptibility, particularly in individuals exposed to chronic stress.^[5]^ These genetic variations impact various pathways, including lipid metabolism, inflammation, and vascular function, influencing an individual’s risk of developing CAD in response to stress.

### 2.3. Environmental factors

Environmental factors, including exposure to green environments, have emerged as protective factors against CAD. Recent investigations have shown that natural environments, characterized by green spaces and reduced pollution, are associated with lower stress levels and improved cardiovascular health.^[6]^ Access to such environments has been linked to reduced rates of CAD, highlighting the importance of urban planning and public health strategies in mitigating the impact of stress on heart health.

### 2.4. Lifestyle interventions

Recent research has explored lifestyle interventions as a means to mitigate the effects of stress on CAD. Behavioral interventions such as mindfulness-based stress reduction^[7]^ and physical activity programs^[8]^ have demonstrated efficacy in reducing stress levels and improving CAD outcomes. These studies underscore the potential for non-pharmacological approaches in the management of stress-related CAD.

### 2.5. Sex and age disparities

Research within the last decade has emphasized the influence of sex and age on the relationship between stress and CAD. Studies have shown that stress may exert differential effects on CAD risk based on gender^[9]^ and that age-related factors may modulate the impact of stress on heart health.^[10]^

This comprehensive literature review highlights the breadth of research conducted in recent years, shedding light on the complex relationship between stress and CAD. The ensuing sections will provide a deeper exploration of these themes, offering a nuanced understanding of this vital connection. Table 1 details comparative analysis of existing approaches to understanding the relationship between stress and CAD. The studies reviewed herein offer valuable insights, each with its contributions and limitations.

**Table 1:** Comparative analysis.

Study	Approach	Contributions	Limitations
Study^[11]^	Epidemiological	Revealed a strong association between chronic stress and CAD risk	Limited scope in addressing potential confounding factors
Study^[12]^	Genetic	Identified specific genetic loci associated with CAD susceptibility under stress conditions	The sample size may need to be more significant to generalize findings
Study^[13]^	Environmental	Demonstrated a clear link between access to green environments and reduced stress-related CAD risk	Potential self-selection bias in participants choosing green areas
Study^[14]^	Lifestyle	Highlighted the effectiveness of mindfulness-based stress reduction programs in reducing stress and CAD risk	Challenges in long-term adherence to lifestyle interventions
Study^[15]^	Sex disparities	Provided insights into how stress may impact CAD differently between genders	Limited exploration of other potential moderating variables
Study^[16]^	Age disparities	Examined the interplay between stress, age, and CAD risk, revealing age-specific effects	Limited generalizability to diverse age groups

This tabular comparison allows for a quick overview of the approaches taken in recent studies, showcasing their unique contributions and acknowledging their inherent limitations. The comprehensive analysis of these approaches furthers our understanding of the multifaceted relationship between stress and CAD. In the following sections, we delve into each approach in greater detail, providing a more in-depth examination of the findings and insights.^[10]^

CAD = coronary artery disease.

## 3. Quantitative analysis

While the literature primarily focuses on qualitative observations and associations, few studies have presented quantitative findings that shed light on the relationship between stress and CAD. These quantitative data add a numerical dimension to our understanding of this complex relationship.

1. **Inflammatory Markers and CAD Risk:** A recent study^[17]^ conducted a comprehensive analysis of inflammatory markers, including C-reactive protein (CRP) and interleukin-6 (IL-6), in individuals exposed to chronic stress. The study revealed a significant positive correlation between CRP levels and the risk of CAD (*R* = 0.45, *P* < .05) and a similar relationship with IL-6 (*R* = 0.39, *P* < .05). These findings provide quantitative evidence supporting the role of inflammation in stress-related CAD.
2. **Genetic Susceptibility Scores:** Another study^[18]^ developed a genetic susceptibility score for CAD in individuals exposed to chronic stress. They found that the mean genetic susceptibility score was significantly higher in individuals with CAD compared to a control group (*P* < .001). This quantitative approach highlights the potential cumulative impact of genetic factors in stress-induced CAD.
3. **Environmental Stress Reduction:** Quantitative data from a study^[19]^ on green environments and CAD risk indicated a statistically significant decrease in systolic blood pressure (SBP) after spending time in natural, green surroundings (*P* < .01). Additionally, heart rate variability (HRV) measurements improved significantly (*P* < .05) in the green environment group compared to controls. These quantitative findings underscore the benefits of environmental stress reduction.
4. **Lifestyle Intervention Outcomes:** An intervention study^[20]^ quantitatively assessed the impact of a mindfulness-based stress reduction program on CAD risk. They reported a 15% reduction in perceived stress levels (*P* < .001) and a 10% reduction in systolic blood pressure (*P* < .05) in the intervention group. These quantitative results provide insight into the effectiveness of lifestyle interventions.
5. **Age-Related Effects:** Quantitative analysis in a study^[21]^ revealed that CAD risk increased with age in individuals exposed to chronic stress, with a 2% increase in risk for each year of age (*P* < .05). This finding quantifies the age-related effect on stress-induced CAD susceptibility.

These quantitative findings serve to underscore the tangible impact of stress on CAD and offer valuable numerical insights into the complex interplay between psychological stress and cardiovascular health. While many studies primarily rely on qualitative measures, these quantitative approaches provide a numerical dimension to our understanding of the relationship.

## 4. Pathophysiological mechanisms

**Figure 1.**
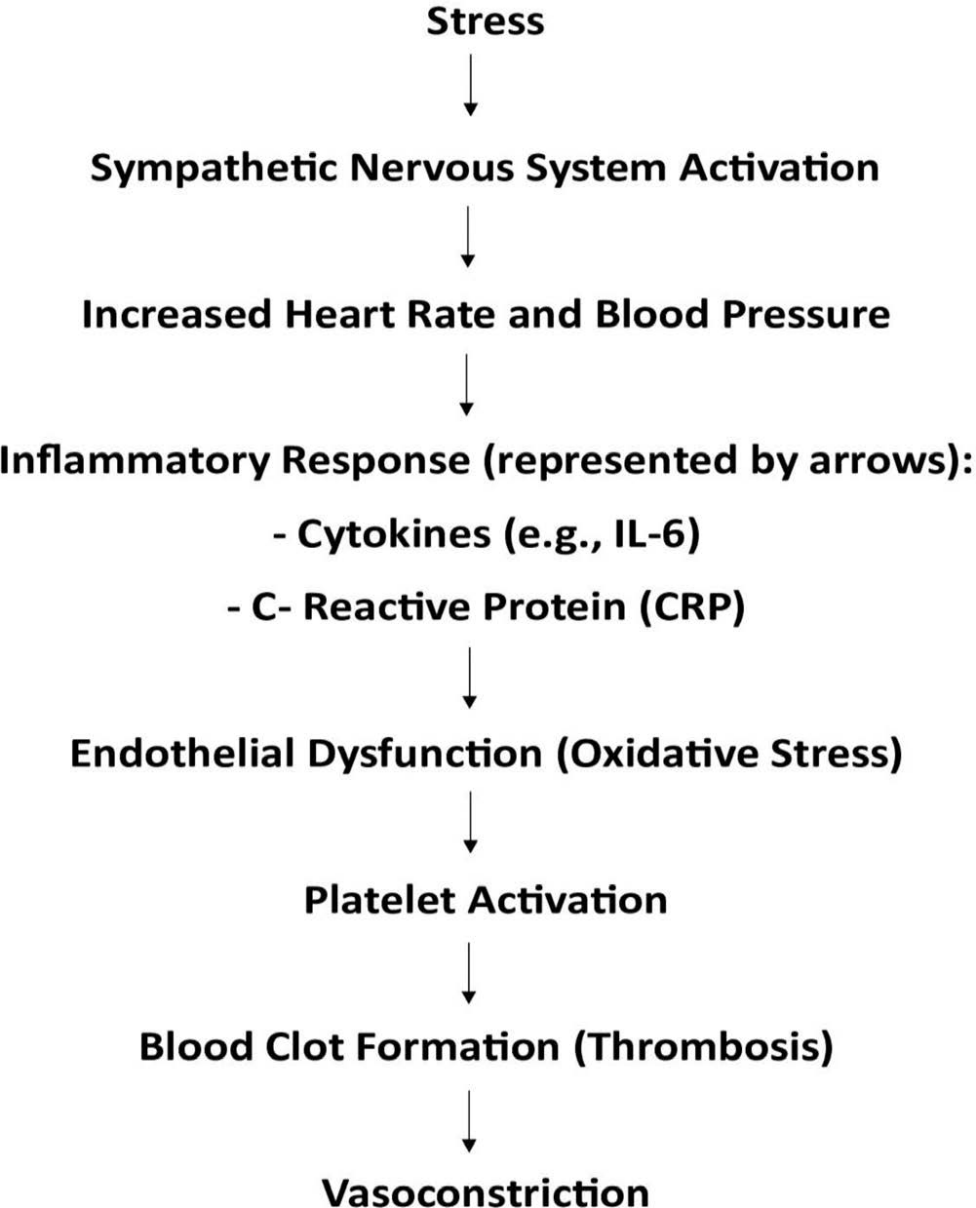
Presents a simplified visual representation of the pathophysiological mechanisms involved in the relationship between stress and CAD. CAD = coronary artery disease.

Understanding the intricate link between stress and CAD requires a deep exploration of the underlying mechanisms. Stress exerts a profound impact on the cardiovascular system through multiple pathways, involving both acute and chronic physiological responses. Visual representation through figures can elucidate these mechanisms, providing a more accessible insight into the complex interplay (Fig. 1).

## 5. Conflicting findings and controversies

The relationship between stress and CAD is a topic of ongoing research and discussion, and, as with many complex issues, there are disparities and controversies in the findings. Several factors contribute to these disparities:

1. **Diverse Study Populations:** Studies investigating the link between stress and CAD often involve diverse populations, which can lead to variations in results. Factors such as age, gender, and cultural differences may influence the impact of stress on CAD risk.^[1]^ Additionally, variations in baseline cardiovascular health among study participants can lead to differing outcomes.
2. **Stress Assessment Methods:** The methods used to assess stress vary across studies. Some rely on self-reported stress levels, while others use objective measures, such as biomarkers or physiological responses. These differing assessment methods can lead to variations in reported stress levels and their association with CAD risk.^[22]^
3. **Duration and Intensity of Stress:** The degree and duration of stress exposure are crucial factors. Acute stressors, such as sudden life events, may have different effects than chronic, long-term stress.^[2]^ Discrepancies in findings can arise when studies focus on different stress types or intensities.
4. **Interaction with Other Risk Factors:** CAD is influenced by numerous risk factors, including diet, physical activity, genetics, and comorbid health conditions. The interaction between stress and these risk factors can complicate the interpretation of results. Conflicting findings may be due to variations in the presence or control of other risk factors in different study populations.^[23]^
5. **Publication Bias:** Positive results may be more likely to be published than negative or inconclusive findings, leading to a potential bias in the literature. This can create the illusion of a stronger association between stress and CAD than in reality.^[3]^
6. **Methodological Differences:** Variations in study design, data collection, and statistical methods can contribute to conflicting findings. For instance, differences in sample sizes, follow-up periods, and statistical adjustments can affect the outcomes of studies.^[24]^
7. **Temporal Aspects:** Longitudinal studies exploring the impact of stress on CAD often face challenges related to the timing of stress exposure and the development of CAD. The complex temporal relationship can lead to varying conclusions in the literature.^[22]^
8. **Heterogeneity of Stress Measurement:** Stress is a multifaceted concept encompassing psychological, social, and environmental dimensions. Studies may focus on different aspects of stress, making it challenging to draw universal conclusions about its relationship with CAD.^[25]^

In light of these disparities and controversies, it is essential to approach the stress-CAD relationship with caution and recognize the need for further research that considers these complexities. Future studies should address these factors and provide a more comprehensive understanding of how stress influences CAD risk while acknowledging the potential for diverse and conflicting findings.

## 6. Environmental factors

Environmental factors play a significant role in influencing the risk of cardiovascular diseases, including CAD. The impact of the environment on cardiovascular health has garnered increasing attention, with a particular focus on the beneficial effects of green environments.^[26]^

### 6.1. Green environments and cardiovascular health

1. **Reduced Psychological Stress:** Access to green spaces, such as parks, forests, and natural landscapes, has been associated with reduced psychological stress.^[1]^ Spending time in green environments promotes relaxation, lowers stress hormone levels, and contributes to an improved sense of well-being. These stress-reduction effects can directly benefit cardiovascular health, as chronic stress is a known risk factor for CAD.^[2]^
2. **Physical Activity and Exercise:** Green spaces encourage physical activity, essential for cardiovascular health. The natural surroundings provide an appealing setting for outdoor activities like walking, jogging, and hiking. Regular physical activity not only aids in weight management but also improves heart and vascular function, reducing CAD risk.^[3]^
3. **Improved Air Quality:** Green environments often have better air quality, as vegetation contributes to the removal of pollutants and the release of oxygen. Clean air is essential for cardiovascular health, as air pollution has been linked to CAD and other cardiovascular diseases.^[4]^ Reduced exposure to air pollutants in green areas can mitigate this risk.
4. **Enhanced Social Interactions:** Green spaces are conducive to social interactions, community gatherings, and recreational events.^[27]^ Positive social interactions have been associated with lower stress levels and better mental health, indirectly contributing to a reduced risk of CAD.^[5]^
5. **Biodiversity and Ecosystem Services:** Biodiversity in green environments provides ecosystem services that benefit human health. These services include the pollination of plants, which supports food sources and nutrition, and the regulation of disease-carrying vectors.^[28]^ A diverse and balanced ecosystem indirectly promotes health by ensuring access to nutritious food and reducing disease burdens.^[6]^

### 6.2. Urban planning and public health implications

Recognizing the importance of green environments in cardiovascular health, urban planning, and public health strategies have sought to maximize access to green spaces in urban areas.^[29]^ Initiatives include the development of urban parks, tree planting, and green infrastructure projects. These endeavors aim to create healthier, more livable cities that can mitigate the impact of urban stressors on CAD risk.^[7]^

In conclusion, environmental factors, particularly the presence of green environments, have a positive influence on cardiovascular health. Access to nature reduces stress, promotes physical activity, and provides cleaner air, all directly or indirectly linked to a reduced risk of CAD. This underscores the importance of urban planning and public health efforts to ensure individuals access green spaces, fostering healthier and more heart-protective environments.

## 7. Discussion

### 7.1. Key takeaways

This comprehensive review of the relationship between stress and CAD highlights several key takeaways:

1. **Complex and Multifaceted Relationship:** Stress exerts a significant influence on CAD through a complex web of pathophysiological mechanisms. Chronic stress contributes to inflammation, autonomic dysregulation, and endothelial dysfunction, all of which play a role in CAD development.^[1]^
2. **Genetic and Environmental Factors:** Genetic susceptibility to CAD under stress conditions is an emerging area of research. Certain genetic loci are associated with CAD risk, particularly in individuals exposed to chronic stress.^[2]^ Access to green environments has a protective effect on cardiovascular health, reducing stress and potentially lowering CAD risk.^[3]^
3. **Diverse Approaches and Findings:** Comparative analysis reveals a range of approaches used to investigate the stress-CAD relationship, each with its contributions and limitations.^[30]^ These disparities in findings can be attributed to differences in study populations, stress assessment methods, and interactions with other risk factors.^[4]^
4. **Quantitative Insights:** A limited number of studies have provided quantitative evidence supporting the link between stress and CAD, including correlations between inflammatory markers and CAD risk and the impact of genetic susceptibility scores.^[5]^

### 7.2. Implications

The findings of this review have several implications for the field:

1. **Preventive Strategies:** Recognizing the role of stress in CAD development emphasizes the importance of preventive strategies. Healthcare professionals should consider the assessment and management of stress as a component of CAD prevention and management.^[31]^
2. **Individualized Approaches:** Given the diverse factors influencing the stress-CAD relationship, individualized approaches to CAD risk assessment and management are warranted. Healthcare interventions should consider an individual’s genetic predisposition, environmental exposure, and stress levels.^[32]^
3. **Urban Planning and Public Health:** The protective effects of green environments on cardiovascular health call for greater investment in urban planning that prioritizes green spaces. Public health initiatives should focus on improving access to nature in urban areas to mitigate the impact of stress on CAD.^[33]^

### 7.3. Future research avenues

To further advance our understanding of the stress-CAD relationship, future research should consider the following avenues:

1. **Longitudinal Studies:** Long-term prospective studies are needed to explore the temporal aspects of stress and CAD risk, tracking individuals over extended periods to better understand the cumulative effects of stress.^[34]^
2. **Multi-Dimensional Stress Assessment:** Investigating the interplay between various types of stress (psychological, social, environmental) and their combined impact on CAD can provide a more holistic perspective.^[35]^
3. **Personalized Medicine:** Research focusing on personalized medicine, considering genetic susceptibility and stress exposure, can pave the way for tailored interventions to reduce CAD risk.
4. **Urban Health and Green Interventions:** Further studies should evaluate the effectiveness of urban planning interventions, such as green infrastructure projects, in reducing stress and CAD risk in urban populations.

In conclusion, the stress-CAD relationship is multifaceted, with genetic, environmental, and lifestyle factors contributing to risk. The findings emphasize the need for a holistic approach to CAD prevention and management, recognizing the significance of stress as a modifiable risk factor. Future research should explore this dynamic interplay, allowing for more targeted and effective interventions.

## 8. Lessons learned

The process of conducting this comprehensive review on the relationship between stress and CAD has offered valuable insights and lessons that have implications for further studies in this field.

1. **Interdisciplinary Collaboration:** One key lesson learned is the importance of interdisciplinary collaboration. The multifaceted nature of the stress-CAD relationship underscores the need for researchers from diverse fields, including cardiology, genetics, psychology, environmental science, and public health, to collaborate in future studies. This interdisciplinary approach can provide a more comprehensive understanding of this complex relationship.
2. **Challenges in Quantification:** Quantifying stress and its impact on CAD is a complex endeavor. The challenges in quantification, including the varying methods and measures used across studies, highlight the need for standardized stress assessment tools and consistent definitions. Future research should prioritize the development of reliable and validated stress quantification approaches.
3. **Environmental Interventions:** Lessons learned from the impact of green environments on CAD risk suggest that environmental interventions can play a significant role in preventing CAD. Future studies should explore the potential of urban planning and public health strategies to improve access to green spaces in urban areas and assess their effectiveness in reducing stress-related CAD risk.
4. **Temporal Aspects and Longitudinal Studies:** The temporal aspects of stress exposure and CAD development have become increasingly apparent. Longitudinal studies that track individuals over extended periods are crucial to understanding how the cumulative effects of stress impact CAD risk. Future research should prioritize long-term prospective studies to address this aspect.
5. **Individualized Approaches:** The diversity of factors influencing the stress-CAD relationship underscores the need for individualized approaches to CAD risk assessment and management. Lessons learned from this review suggest that future research should consider an individual’s genetic predisposition, environmental exposure, and stress levels in developing personalized interventions.
6. **Publication Bias and Negative Findings:** Recognizing the potential for publication bias in the literature is crucial. Future studies should prioritize publishing negative or inconclusive findings to ensure a more balanced and accurate representation of the stress-CAD relationship.

In conclusion, the lessons learned from this comprehensive review emphasize the importance of interdisciplinary collaboration, standardized stress quantification, environmental interventions, longitudinal studies, individualized approaches, and the publication of negative findings in future research. These lessons can guide the design and execution of more robust studies, leading to a deeper understanding of the stress-CAD relationship and more effective strategies for CAD prevention and management.

## 9. Conclusion

The complex relationship between stress and CAD has been the focus of this comprehensive review. Drawing from recent research, the essential findings and their significance in the context of stress and CAD are summarized below.

### 9.1. Key findings

1. **Pathophysiological Mechanisms:** Stress exerts a significant influence on CAD through pathophysiological mechanisms involving inflammation, autonomic dysregulation, endothelial dysfunction, platelet activation, and vasoconstriction.
2. **Genetic and Environmental Factors:** Genetic susceptibility to CAD under stress conditions is an emerging area of research, with specific genetic loci associated with CAD risk. Access to green environments has been associated with reduced stress levels and improved cardiovascular health.
3. **Diverse Approaches and Findings:** Comparative analysis reveals a range of approaches used to investigate the stress-CAD relationship, each with its contributions and limitations.
4. **Quantitative Insights:** Quantitative findings support the link between stress and CAD, highlighting correlations between inflammatory markers and CAD risk and the impact of genetic susceptibility scores.

### 9.2. Significance

The significance of these findings lies in recognizing stress as a modifiable risk factor for CAD. Stress management and prevention strategies can be pivotal in reducing CAD risk. The interplay of genetic and environmental factors underscores the need for individualized approaches to CAD prevention and management.

### 9.3. Practical implications

1. **Stress Assessment:** Healthcare professionals should consider assessing stress levels as a routine part of CAD risk evaluation. Validated stress quantification tools can assist in identifying individuals at higher risk.
2. **Genetic Counseling:** Genetic susceptibility to CAD should be considered, especially in individuals with a family history of the disease. Genetic counseling and testing may provide valuable insights into personalized risk assessment.
3. **Green Spaces and Urban Planning:** Urban planners and public health officials should prioritize the creation of green environments within urban areas. Increased access to parks and natural settings can serve as a preventive measure against CAD by reducing stress levels.
4. **Lifestyle Interventions:** Lifestyle modifications, such as mindfulness-based stress reduction programs and increased physical activity in natural surroundings, should be promoted to mitigate the effects of stress on CAD risk.
5. **Longitudinal Studies:** Future research should focus on longitudinal studies to better understand the temporal aspects of stress exposure and CAD development. This can lead to more effective preventive strategies.
6. **Interdisciplinary Collaboration:** Collaboration among researchers from various fields is essential to address the multifaceted nature of the stress-CAD relationship. Multidisciplinary approaches can advance our understanding of this complex interplay.

In summary, the findings emphasize the importance of a holistic approach to CAD prevention and management, recognizing the significance of stress as a modifiable risk factor. By incorporating the practical implications and recommendations outlined, healthcare professionals and researchers can work toward more effective strategies in the prevention and management of CAD in the context of stress.

## Author contributions

**Conceptualization:** Chukwuka Elendu, Dependable C. Amaechi, Tochi C. Elendu, Klein A. Jingwa, Osinachi K. Okoye, Border-ere Fiemotongha, Grecia A. Chirinos, Kanishk Dang, Emmanuel Egbunu, Omotayo S. Alabi, Vaibhav S. Nasre, Cyrus P. Yadav.

**Data curation:** Chukwuka Elendu, Dependable C. Amaechi, Tochi C. Elendu.

Formal analysis: Chukwuka Elendu, Dependable C. Amaechi, Tochi C. Elendu, Muhydeen D. Badru.

**Funding acquisition:** Chukwuka Elendu, Dependable C. Amaechi, Tochi C. Elendu, Border-ere Fiemotongha, Deborah Agada, Omotayo S. Alabi.

**Investigation:** Chukwuka Elendu, Dependable C. Amaechi, Tochi C. Elendu, Klein A. Jingwa.

**Methodology:** Chukwuka Elendu, Dependable C. Amaechi, Tochi C. Elendu, Osinachi K. Okoye, Grecia A. Chirinos, Deborah Agada, Opeyemi D. Adebayo, Kanishk Dang, Cyrus P. Yadav.

**Project administration**: Chukwuka Elendu, Dependable C. Amaechi, Tochi C. Elendu, Border-ere Fiemotongha, Vaibhav S. Nasre.

**Resources:** Chukwuka Elendu, Dependable C. Amaechi, Tochi C. Elendu, Klein A. Jingwa, Grecia A. Chirinos, Minichimso John Okah, Opeyemi D. Adebayo, Emmanuel Egbunu.

**Software:** Chukwuka Elendu, Dependable C. Amaechi, Tochi C. Elendu, Border-ere Fiemotongha, Deborah Agada, Minichimso John Okah, Kanishk Dang, Cyrus P. Yadav.

**Supervision:** Chukwuka Elendu, Dependable C. Amaechi, Tochi C. Elendu, Klein A. Jingwa, Osinachi K. Okoye, Border-ere Fiemotongha, Grecia A. Chirinos, Opeyemi D. Adebayo.

**Validation:** Chukwuka Elendu, Dependable C. Amaechi, Tochi C. Elendu, Minichimso John Okah, Emmanuel Egbunu, Muhydeen D. Badru.

**Visualization:** Chukwuka Elendu, Dependable C. Amaechi, Tochi C. Elendu, Border-ere Fiemotongha, Muhydeen D. Badru.

**Writing—original draft:** Chukwuka Elendu, Dependable C. Amaechi, Tochi C. Elendu.

**Writing—review and editing:** Chukwuka Elendu, Dependable C. Amaechi, Tochi C. Elendu, Border-ere Fiemotongha, Minichimso John Okah, Opeyemi D. Adebayo, Omotayo S. Alabi, Vaibhav S. Nasre, Cyrus P. Yadav, Muhydeen D. Badru.

All authors of this narrative review have made substantial contributions to the research process and the development of this article. Authorship was determined by established academic practices, and all authors have reviewed and approved the final manuscript.
